# Exploring the Efficacy of Combining Radiofrequency Thermal Ablation or Microwave Ablation with Vertebroplasty for Pain Control and Disease Management in Metastatic Bone Disease—A Systematic Review

**DOI:** 10.3390/curroncol31090401

**Published:** 2024-09-13

**Authors:** Eliodoro Faiella, Federica Vaccarino, Giuseppina Pacella, Domiziana Santucci, Elva Vergantino, Amalia Bruno, Raffaele Ragone, Bruno Beomonte Zobel, Rosario Francesco Grasso

**Affiliations:** 1 Operative Research Unit of Radiology and Interventional Radiology, Fondazione Policlinico Universitario Campus Bio-Medico di Roma, Via Alvaro del Portillo 200, 00128 Rome, Italy; 2 Research Unit of Radiology and Interventional Radiology, Department of Medicine and Surgery, Università Campus Bio-Medico di Roma, Via Alvaro del Portillo 21, 00128 Rome, Italy

**Keywords:** radiofrequency thermal ablation, microwave ablation, metastatic bone, vertebroplasty, vertebral augmentation

## Abstract

Background: Interventional radiology techniques have become pivotal in recent years in managing metastatic bone disease, which frequently results in skeletal complications such as fractures and severe pain. Thermoablative methods like radiofrequency ablation (RFA) and microwave ablation (MWA), when combined with vertebroplasty (VP), are proving increasingly beneficial for these patients. Methods: The search was independently conducted by two radiologists on MEDLINE databases, using specified strings up to April 2024. Methodological quality was assessed using PRISMA guidelines. Studies meeting inclusion criteria investigated thermoablation techniques (RFA and/or MWA) combined with VP, focusing on pain management and disease control outcomes in adults. Results: Among 147 results, 42 articles met the criteria, with varied prospective and retrospective designs and sample sizes averaging 49 patients, predominantly involving RFA (30 studies), MWA (11 studies), and one comparative study. Our review highlights significant pain reduction, effective local tumor control, and favorable safety of combined RFA or MWA with VP, supporting its potential in managing vertebral pathologies and warranting further clinical integration. Conclusions: The combined treatment of RFA/MWA with VP demonstrates significant pain reduction and local tumor control, with a rapid onset of analgesic effect. These findings support its crucial role in clinical practice for managing vertebral metastases.

## 1. Introduction

Metastatic bone disease predominantly occurs in association with specific cancer types, especially breast cancer (70%), prostate cancer (85%), lung cancer (40%), and kidney cancer (40%). Bone involvement is also observed in 95% of cases of multiple myeloma (MM) [1]. Bone metastases commonly affect the axial skeleton, often leading to skeletal-related events such as pathological fractures, which subsequently result in bone pain. Back pain from vertebral fractures is especially common, affecting about three-quarters of multiple myeloma patients at the time of diagnosis. These events lead to reduced mobility, impaired social functioning, compromised quality of life, increased healthcare costs, and worse survival outcomes [1,2]. In the most severe cases, they can lead to acute spinal cord compression, which, regardless of the cause, can cause swelling and decreased blood flow, potentially leading to permanent neurological damage if not addressed promptly [3]. To evaluate soft tissue involvement or extramedullary disease, a comprehensive MRI study with gadolinium-based contrast agents may be necessary for a more thorough assessment [4].

The primary goals of treating bone metastases are to halt disease progression and alleviate symptoms. The specific treatment approach depends on the specific patient and primary tumor characteristics.

Over recent decades, interventional radiology (IR) has notably expanded its role in bone tumor management, in particular in bone metastases, which stand as the most prevalent form of bone tumor [5,6]. IR techniques used to manage bone metastases can be divided into “consolidative methods”, which focus on stabilizing or preventing fractures without ensuring effective tumor control, and “ablative techniques”, involving tumor destruction, acting as radical treatment if complete destruction is achieved or as palliative measures, which nonetheless allow significant pain relief [7]. Choosing the most suitable IR technique relies on various factors, such as the biomechanics of the affected bone, its radiographic appearance, and the nature of the pain experienced (either ‘mechanical’ pain, alleviated by rest, or ‘inflammatory’ pain, persistently present due to mediators released by the bone metastasis itself). Thermoablative techniques, a type of ablative procedure, cause coagulation necrosis in the target tissue by using specialized applicators (such as antennas, electrodes, and probes), which are positioned in the tumor via image guidance. These methods include radiofrequency ablation (RFA) and microwave ablation (MWA) [8].

Among consolidation techniques, osteoplasty is preferred when the bone is primarily subjected to axial loads, such as the acetabular roof or the vertebral body [9]. Percutaneous vertebroplasty (VP) represents a minimally invasive approach for addressing symptomatic vertebral compression fractures. It functions by enhancing spinal stability and leveraging the analgesic properties of biocompatible cement due to the thermal and chemical effect of the cement on nociceptors [10]. The procedure involves injecting bone cement, typically polymethylmethacrylate (PMMA), into the target bone. This is done under continuous fluoroscopic guidance, using one or more bone trocars. The goal of cement injection is to maximize filling of the bone defect. Research indicates that optimal cement filling notably decreases the likelihood of secondary fractures or worsening of existing fractures [11]. Studies in the literature have explored the combination of ablative treatments, in particular RFA or MWA, with VP. This review aims to summarize and analyze these studies to determine the effectiveness of combined approaches for pain management and tumor control. By synthesizing these findings, we seek to provide insights into the optimal use of combined ablative treatments and VP in clinical practice.

## 2. Materials and Methods

A comprehensive literature search was conducted independently by two different radiologists in electronic databases, including PubMed, Scopus, and Web of Science, using the following strings: (“Thermoablation” OR “Thermo Ablation” OR “Microwave Ablation” OR “MWA” OR “RFA” OR “radiofrequency ablation” OR “percutaneous ablation” OR “image guided ablation”) AND (“vertebroplasty” OR “vertebral augmentation” OR “vertebral cementoplasty”). The search strategy was conducted without applying any limitations. The last update of the search was conducted up to April 2024. The methodological quality of included studies was assessed using the PRISMA guidelines and checklist, and the flowcharts of included studies were adhered to. The PRISMA checklist is available in the Supplementary Materials.

### 2.1. Inclusion and Exclusion Criteria

Included studies met the following criteria: investigations into the combination of thermoablation techniques (RFA and/or MWA) with VP, reporting outcomes related to pain management and/or disease control, conducted on populations aged 18 years or older, and published in English. The following types of studies were excluded: abstracts, reviews, letters to editors, case reports, and editorials; studies not focusing on the combination of thermoablation and VP; studies utilizing this combination in skeletal segments other than the vertebrae or those treating benign lesions; and research not available in English. Additionally, studies involving animals were excluded.

### 2.2. Data Extraction

Two independent reviewers screened the titles and abstracts of the retrieved articles to assess their eligibility based on the inclusion and exclusion criteria. Full-text articles of potentially eligible studies were then retrieved and assessed for final inclusion. In the data collection process for each study, the following information was gathered: title, authors, publication date, number of patients, study design (prospective or retrospective), follow-up period (in days), details of the treatment performed (MWA/RFA), details of the vertebroplasty technique, type of imaging guidance used, navigation system (if applicable), previous treatments received (e.g., chemotherapy, radiation therapy, interventional therapy), pain scale used (e.g., VAS/BPI/RMQ), baseline pain level, post-operative pain level, time point of post-operative pain measurement, change in quality of life, and any complications reported.

## 3. Results

**Figure 1:**
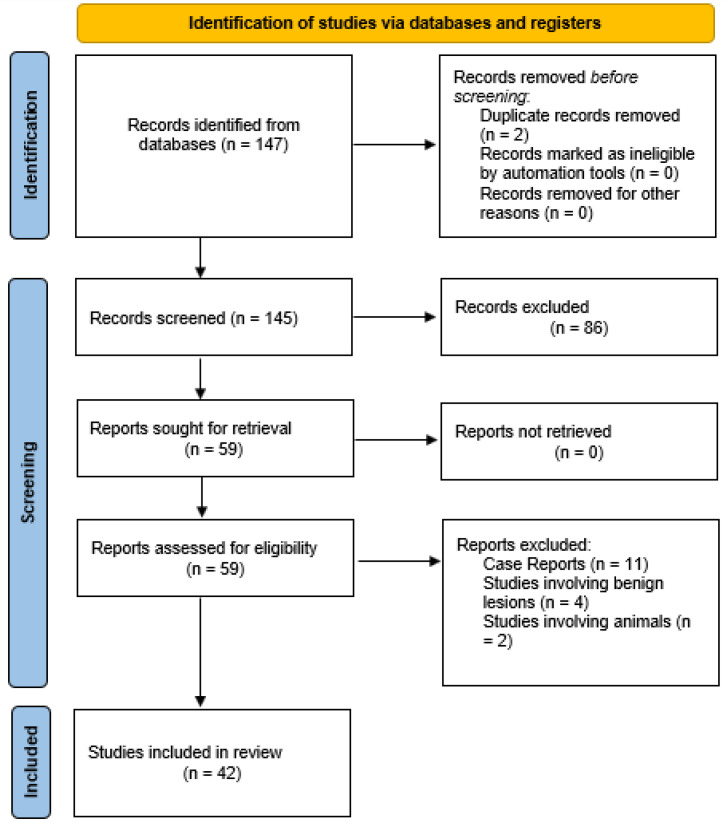
PRISMA 2020 flow diagram for the selection of studies included in the review.

Out of 147 results, 42 articles met the inclusion criteria, while 105 were excluded. Among the excluded articles, 37 were reviews, 1 was a book, 1 was a duplicate article in Spanish, 11 were case reports, 1 was a letter to the editor, 4 articles addressed benign lesions, and 2 articles involved animal studies (the flow diagram for the selection of the studies included is summarized in Figure 1).

The included articles ranged in publication dates from 2007 to 2024. Eight of them are prospective studies, while the remainder are retrospective. The sample sizes varied considerably across the studies, ranging from 11 to 169 patients, with an average of approximately 49 patients.

Out of the 42 included articles, 30 involve RFA, 11 MWA, and 1 compares both techniques. Most of the procedures were performed using both fluoroscopic and CT guidance, with the majority conducted by interventional radiologists.

### 3.1. RFA and VP

Table 1 summarizes all the studies included in the review on the combination of RFA and VP, highlighting their best outcomes.

The number of patients varies significantly across studies, ranging from a minimum of 11 patients (Cazzato et al. [12]) to a maximum of 169 patients (Lu et al. [13]).

- Pain Assessment: pain assessment was primarily conducted using the Visual Analog Scale (VAS), with some studies employing other measures such as the Numeric Rating Scale (NRS), Brief Pain Inventory, and other specific scales.
- Pain Outcomes: across the studies, there is a consistent trend of significant pain reduction following the procedures. For instance, in studies using VAS, the pain scores typically decreased from high levels (e.g., 7–9) before the procedure, to lower levels (e.g., 1–3) within weeks to months post procedure. This suggests a substantial immediate and sustained benefit in pain relief.
- Tumor Control: tumor control was not consistently reported across all studies, making a direct comparison challenging. However, some studies reported specific outcomes. Tomasian et al., indeed, reported a local tumor control rate of 78.9% [14]. Wallace et al. found local tumor control rates of 89% at the 3-months follow-up, decreasing to 70% at 1 year post treatment [15].
- Progression-Free Survival: Yang et al. [16] noted that 66.67% of patients did not show tumor progression, with a mean progression-free survival of approximately 330 days.
- Functional Outcomes and Quality of Life: some studies utilized functional measures like the Oswestry Disability Index (ODI) and the Karnofsky Performance Scale (KPS) to assess the impact on patients’ daily activities and overall well-being. These studies reported significant improvements post procedure; studies that included quality-of-life assessments, such as those using the European Organization for Research and Treatment of Cancer QLQ-C30 questionnaire, showed notable improvements post intervention (e.g., Zhang et al. [17]), indicating that pain relief is often accompanied by enhanced functional status and quality of life.
- Complication and Safety: most studies reported low complication rates. For example, Tomasian et al. noted a total complication rate of 3.0%, with major complications at 0.4%. This suggests that the procedures are generally safe, with minimal risk of severe adverse effects [14].
- Specific Risks: some of these studies identified specific risk factors related to procedures. Wang et al. highlighted risk factors for pulmonary cement embolism, such as multiple segment involvement, thoracic vertebrae location, and the unipedicular puncture approach [18].
- Overall Analysis: the consistent reduction in pain scores and improvements in functional and quality-of-life measures across multiple studies indicate that the combination of RFA and VP provides substantial benefit to patients.

### 3.2. MWA and VP

All studies included in the review on the combination of MWA and VP and their outcomes are summarized in Table 2.

Most studies were retrospective, with patient numbers varying from small cohorts (e.g., Motaghi et al. [19] with 28 patients) to larger ones (e.g., Fan et al. [20] with 38 patients and Wu et al. [21] with 71 patients).

Similarly, available studies consistently show superior outcomes for MWA combined with VP, particularly in pain management (calculated with VAS, NRS, and other pain scores), as well as in the ODI, and in reduced morphine consumption. Baseline VAS scores typically range from 6 to 8, and post-treatment VAS scores generally decrease across all studies, with some dropping as low as 0.6 at 12 months, as reported by Pusceddu et al. [22]. The median VAS score at the last follow-up is approximately 2.5 to 3 in studies with follow-up periods beyond six months, with a range of post-treatment VAS scores from 0.6 to 3.5 at the final follow-up.

Tumor control was a significant outcome measure in several studies. For instance, Wu et al. [21,23] reported no local tumor progression on follow-up imaging, indicating effective local control of the disease. Liu et al. [24] noted lower tumor progression rates in patients receiving the combined treatment compared to VP alone, suggesting that the addition of MWA contributes to better tumor control. Pusceddu et al. [22] and Xiang et al. [25] reported no local disease recurrence during follow-up.

### 3.3. MWA vs. RFA

In terms of comparing the combination of MWA and VP with RFA and VP, the studies in the literature are rare. Positive outcomes for both combinations were highlighted in a 2023 multicenter study conducted by Alfonso M. et al., aimed at evaluating their efficacy in the management of metastatic vertebral fractures. The pain improvement outcome using both ablation methods is very similar, with VAS values decreasing from 7.7 to 2.6 at 6 weeks in the group treated with RFA, and from 6.8 to 1.7 at 6 weeks in the group treated with MWA [26].

**Table 1:** Studies investigating the combined approach of RFA and vertebroplasty, with their summarized best outcomes.

Authors	Year	Study Design	Number of Patients	Mean Age	Pain Score and Other Scores Used	Tumor Control	Best Results
Orgera et al. [27]	2014	Prospective	36	66	VAS and RMQ	n/s	VAS Score (Mean):
							Before procedure: 9.1
							After 24 h: 3.4
							After 6 weeks: 2.0
							RMQ Score (Mean):
							Before procedure: 19.8
							After 24 h: 9.6
							After 6 weeks: 8.2
Alfonso et al. [26]	2023	Retrospective	14	67	VAS	n/s	VAS score (mean):
							Before procedure: 7.7
							After 6 weeks: 2.6
Senol et al. [28]	2022	Retrospective	41	67	VAS and ODI	n/s	VAS score (mean):
							Before procedure: 7.4
							After 1 week and 1 months: 2.5
							After 6 months: 3.2
							ODI score (mean):
							Before procedure: 71.08
							Postoperative 30.1
Tian et al. [29]	2022	Retrospective	126	54	VAS, ODI and KPS	n/s	VAS score (mean):
							Before procedure: 7.43
							After 24 h: 2.25
							VAS, ODI and KPS showed significant improvement after treatment (*p* < 0.05).
Wallace et al. [30]	2015	Retrospective	72	68	NRS	n/s	NRS score (mean):
							Before procedure: 8
							After 1 week: 3.25
							After 4 week: 2.75
Yang et al. [16]	2017	Retrospective	25	57	KPS, ODI, VAS	A total of 66.67% patients did not present tumor progression. The mean progression-free survival: 330 ± 54 days.	VAS values were significantly reduced after surgery.
Tomasian et al. [14]	2021	Retrospective	166	n/s	Brief Pain Inventory scores	local tumor control rate: 78.9%	total complication rate: 3.0%;
							major complication rate: 0.4%;
							minor complication rate: 2.6%
Cazzato et al. [12]	2018	Retrospective	11	61	VAS and Wilcoxon test	n/s	Mean pain score:
							baseline: 7.8 ± 1.1;
							last clinical follow-up available (mean 1.9 ± 1.4 months): 3.5 ± 2
Wang et al. [31]	2022	Retrospective	15	62	VAS	n/s	VAS scores in the group that used the combination of RFA and VP decreased more rapidly one week after the treatments and remained more stable at six months compared to the group that underwent VP alone.
Wang et al. [18]	2023	Retrospective	47	60	VAS	n/s	Risk analysis showed that multiple segments (≥3, *p* = 0.022), thoracic vertebrae (*p* = 0.0008), and unipedicular puncture approach (*p* = 0.0059) are risk factors for pulmonary cement embolism.
Yildizhan et al. [32]	2021	Retrospective	40	n/s	VAS and ODI	No patients developed spine fractures or cord compression from tumor spread during the follow-up.	VAS Score (mean): Before procedure: 7.44 ± 1.06 in group RFA + VP; the difference in the mean VAS scores was statistically significant at all measurement time-points after the procedure (*p* < 0.001). Pre-treatment ODI (mean): 78.50% in the RFA + VP group, which improved to 14.2% after treatment.
Zhang et al. [17]	2020	Retrospective	15	50	VAS, Frankel grade, European Organization for Research and Treatment of Cancer (EORTC) QLQ-C30 questionnaire	n/s	Pre-op VAS (mean): 7.86 ± 0.86
							Post-op (3 months) mean VAS: 3.51 ± 1.32
							Frankel grade: improved 1–2 grades in 14/15 patients
							EORTC QLQ-C30 score: pre-op 86.13 ± 8.51, post-op 52.21 ± 13.28
Sayed et al. [33]	2019	Prospective	30	>18	NRS and Functional Assessment of Cancer Therapy-General 7 (FACT-G7)	n/s	Average NRS-11 scores decreased from a baseline of 5.77 to 4.65 (3 days), 3.33 (one week), 2.64 (one month), and 2.61 (3 months).
							FACT-G7 increased from a baseline average of 13.0 to 14.7 (3 days), 14.69 (one week), 14.04 (one month), and 15.11 (3 months).
Tomasian et al. [34]	2018	Retrospective	27	55	n/a	Local tumor control was achieved in 96% of tumors, according to median imaging follow-up of 16 weeks.	Simultaneous bipedicular RFA combined with VP is safe and effective for local tumor control of vertebral metastases.
Pusceddu et al. [35]	2021	Retrospective	35	59	VAS	No tumor recurrence in index vertebrae at median follow-up of 19 months (range 4–46 months) and 10 months (range 4–37 months).	Baseline VAS score decreased significantly from 5.7 (95% CI 4.9–6.5) before tRFA to 0.9 (95% CI 0.4–1.3) after tRFA. The mean decrease in VAS score from baseline to one-week follow-up was 4.8 (95% CI 4.2–5.4).
Giammalva et al. [36]	2022	Retrospective	54	63	VAS	n/a	The average baseline VAS score significantly decreased from 7.81 to 2.50 after 12 months.
Zhao et al. [37]	2018	Prospective	16	69	VAS, EORTC and QLQ-C30	n/a	Baseline VAS score: 8.1 ± 1.4, and reduced significantly to 5.5 ± 1.1 at 24 h, 2.8 ± 0.6 at 1 week, and 1.4 ± 0.8 at 6 months (*p* < 0.01).
							EORTC QLQ-C30 scale at 1 month showed significant improvement.
Madani et al. [38]	2022	Retrospective	18	54	VAS	n/a	Baseline VAS score decreased from 7.3 ± 2.4 to 2 ± 0 (*p* = 0.008), along with a decrease in the mean morphine milligram-equivalent dose from 196.6 ± 135.7 to 38.5 ± 26 (*p* = 0.008).
Kastler et al. [39]	2021	Retrospective	25	60	VAS	n/a	Post-procedure follow-up mean VAS score decreased by 74% at day 1 (6.6, *p* < 0.001), 79% at 1 month (6.6, *p* < 0.001), 79% at 3 months (6.5, *p* < 0.001), 77% at 6 months, and 79% at 12 months (6.6, *p* < 0.001).
Wallace et al. [30]	2016	Retrospective	55	n/a	n/a	Radiographic local tumor control rates were 89% (41/46) at 3 months, 74% (26/35) at 6 months, and 70% (21/30) at 1 year post treatment.	The combined approach of RFA and VP shows effectiveness in achieving local control of spinal metastases.
He et al. [40]	2021	Retrospective	40	n/a	VAS and spinal stenosis rates (SSRs)	n/a	VP combined with RFA shows short-term pain relief advantages, whereas VP combined with 125I particle implantation may offer superior pain relief and reduce SSRs at 3 months post operation.
Pusceddu at al. [41]	2023	Retrospective	16	67	VAS and Functional Mobility Scale (FMS)	n/a	A statistically significant reduction in VAS score was observed before treatment and 1 week after treatment. The improvement in mobility before and after treatment was statistically significant.
David et al. [42]	2017	Retrospective	26	72	n/a	n/a	Using RFA prior to VP significantly reduced the rate of posterior and venous cement leaks. Pain scores decreased significantly post procedure.
Reyes et al. [43]	2018	Retrospective	49	n/a	VAS and ODI	n/a	Baseline VAS scores (mean) decreased from 7.9 ± 2.5 pre-procedure to 3.5 ± 2.6 post procedure. Mean ODI scores improved from 34.9 ± 18.3 to 21.6 ± 13.8 post procedure.
Lu et al. [13]	2019	Retrospective	169	57	VAS, WHO Pain Relief and ODI	n/a	VP combined with 125I Seed exhibited the best clinical efficacy in terms of VAS score. VP combined with RT was the best in terms of WHO Pain Relief. VP combined with RFA showed the best effect in terms of ODI.
Maugeri et al. [44]	2017	Retrospective	18	56	VAS	n/a	Baseline VAS score (mean): decreased significantly from 8.05 to 3.0 (*p* < 0.05) after 6 months.
Clarençon et al. [45]	2013	Prospective	24	61	VAS	Local recurrence: 6/24 cases	Mean VAS was 1.9 (±2.4) at 1 month FU, and 2.3 (±2.9) at 6 months’ FU. Pain was significantly reduced at 6 months’ FU (mean VAS reduction = 4.1; *p* < 0.00001).
Pezeshki et al. [46]	2016	n/a	12	n/a	n/a	n/a	The combination of RFA and cement placement in the posterior vertebral body shows markedly enhanced vertebral stability when subjected to axial loading.
Van der Linden et al. [47]	2007	Retrospective	12	57	VAS	n/a	Baseline VAS score (mean): 17.33 ± 2.46 (range, 13–20) versus 9.25 ± 4.81 (range, 2–18) one week after treatment (*p* < 0.001) and 7.00 ± 5.26 (range, 1–14) three months after treatment (*p* = 0.020).
Hoffmann et al. [48]	2008	Retrospective	22	64	VAS	n/a	Baseline VAS score (mean): 8.5, which decreased to a mean of 5.5 after 24 h (*p* < 0.01), and a further decrease was detected after 3 months, to 3.5 (*p* < 0.01).
Song et al. [49]	2014	Prospective	12	59	VAS and KPS	n/a	Baseline VAS score (mean): 7.0 1.0, which decreased to 2.1 ± 1.2 at 1 month, to 1.6 ± 1.4 at 6 months, to 1.8 ± 1.7 at 1 year, and was maintained at 1.3 ± 1.1 at >1-year follow-up. KPS improving from 64.17 ± 8.20 preoperatively, to 66.58 ± 5.53 at 1 week, to 68.24 ± 3.60 at 1 month, and 68.25 ± 5.35 at 3 months, to 68.83 ± 5.86 at 6 months, to 67.13 ± 7.12 at 1 year, to 70.33 ± 8.14 at >1 year.

**Table 2:** Studies investigating the combined approach of MWA and VP, with their summarized best outcomes.

Authors	Year	Study Design	Number of Patients	Pain Score and Other Scores Used	Tumor Control	Best Results
Chen et al. [50]	2022	Retrospective	91	VAS, daily morphine consumption, ODI	n/s	Baseline (mean) VAS: 6, Morphine: 77.8 mg
						Post procedure:
						3 days: VAS: 5, Morphine: 34.5 mg
						1 week: VAS: 4, Morphine: 28.7 mg
						1 month: VAS: 3, Morphine: 24.6 mg
						3 months: VAS: 3, Morphine: 21.7 mg
						6 months: VAS: 3, Morphine: 21.0 mg
Hu et al. [51]	2022	Prospective	67	VAS, analgesic use scores (AUS), and quality-of-life score (QLS)	n/s	The VAS score was lower at 6 months (2.7 ± 0.7 vs. 3.2 ± 0.7) and 12 months (3.5 ± 0.8 vs. 4.0 ± 0.7). The AUS and QLS were improved at 12 months (*p* < 0.05).
Fan et al. [20]	2023	Retrospective	38	VAS, daily morphine consumption and ODI	n/s	Baseline VAS score (mean): 6.40 ± 1.90; which decreased to 3.32 ± 0.96 at 24 h, 2.24 ± 0.91 at 1 week, 1.92 ± 1.32 at 4 weeks, 1.79 ± 1.45 at 12 weeks, and 1.39 ± 1.12 at 24 weeks postoperatively. In the follow up period, the ODI and morphine consumption significantly reduced.
Wu et al. [23]	2021	Retrospective	23	VAS, daily morphine consumption and ODI	No local tumor progression in Follow-up imaging.	mean VAS scores, morphine consumption and ODI scores significantly decreased after treatment.
Liu et al. [24]	2023	Retrospective	58	VAS	Tumor progression was lower in the combined-treatment group compared to VP alone (33.00% vs. 7.14%, *p* = 0.02).	MWA combined with PVP resulted in more sustained pain relief (>6 months) and ultimately improved quality of life with lower tumor progression and cement leakage rates, compared to PVP alone.
Wang et al. [52]	2024	Retrospective	20	VAS, ODI and Quality of Life Questionnaire–Bone Metastases 22 (QLQ-BM22)	Local recurrence occurred in only three patients.	Baseline VAS scores (mean): 7.25 ± 0.91.
						After 1 day: 3.70 ± 1.12;
						After 1 week: 2.70 ± 0.7;
						After 1 months: 2.40 ± 0.68;
						After 3 months: 2.25 ± 0.71;
						After 6 months: 2.70 ± 0.92.
						All values were significantly lower. ODI and QLQ-BM22 significantly lower after procedure.
Wu et al. [21]	2024	Retrospective	71	VAS, daily morphine-equivalent opioid consumption and ODI	No local tumor progression on follow-up imaging.	VAS, daily morphine-equivalent opioid consumption and ODI values significantly lower after treatment.
Pusceddu et al. [22]	2023	Retrospective	16	VAS and ODI	No local disease recurrence was reported during a median follow-up period of 12 months.	Baseline VAS score (mean): 6.8 ± 0.7, which decreased to 0.6 ± 0.6 after treatment. ODI score decreased from 3.1 ± 0.7 to 1.2 ± 0.4.
Pusceddu et al. [53]	2023	Retrospective	28	VAS and Functional Mobility Scale (FMS)	No local disease recurrence was reported at MRI performed 6 months after the procedure.	Combining MWA with bilateral expandable titanium SpineJack implants and VP is safe and effective, stabilizes vertebrae, relieves pain early and persistently, enhances mobility, improves walking recovery, and achieves local tumor control.
Motaghi et al. [19]	2022	Retrospective	28	VAS	At PET/CT scan: median pre-procedure SUVmax was significantly reduced following ablation, from 4.55 to 0 over an average of 29 ± 14.1 month follow-up period.	Baseline VAS score (mean): 8 (6.5–9), which decreased to 1(1–2), 2(1–3) and 1(0.5–3) at 24 h, four weeks, and six months post procedure, respectively.
Xiang et al. [25]	2020	Retrospective	12	VAS and ASIA neurologic grading system	Local tumor recurrence in 1 patient during follow up period.	VAS scores were 2.7 ± 0.6, 2.5 ± 0.4, 2.6 ± 0.5, and 2.5 ± 0.5 at 1, 3, 6 months after surgery and at final follow-up (significantly improved compared with baseline).

## 4. Discussion

**Figure 2:**
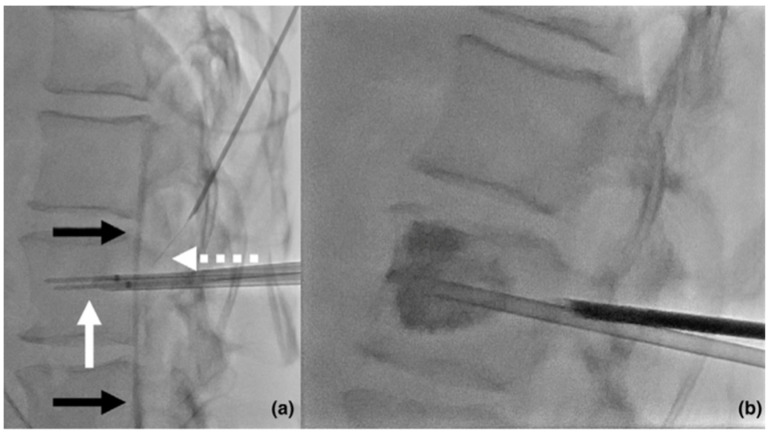
(**a**) Sagittal fluoroscopic image showing a bipolar radiofrequency ablation procedure to achieve local tumor control of an L1 lung cancer metastasis in a 52-year-old oligometastatic female patient; the intervention was carried out with two cooled electrodes (solid white arrow) deployed trans-pedicularly through two different bone trocars. Moreover, a coaxial 18 G needle was used to provide thermal monitoring (dotted arrow) and protective hydrodissection of the anterior epidural space (black arrows). (**b**) Sagittal fluoroscopic image showing how, following ablation, VP was performed in the vertebral body to prevent a secondary fracture.

RFA employs an applicator to administer high-frequency alternating current (typically between 400 and 500 kHz) to the target tissue, resulting in ionic agitation and frictional heat generation, elevating temperatures to 60–100 °C. The extent of the ablation zone achieved is contingent upon tissue impedance, as well as adjacent perfusion and ventilation. MWA, conversely, utilizes an electrical current from a 915-MHz or 2.45-GHz generator, transmitted through a water-cooled interstitial antenna. This generates a local non-ionizing electromagnetic field that interacts with dipolar molecules within the tissue, inducing frictional heating [5]. The results analyzed by this review show that both RFA and MWA, when combined with VP, show better outcomes overall in terms of pain management and tumor control, and provide greater vertebral stability, thereby preventing potential collapses and/or fractures (Figure 2).

### 4.1. RFA and VP

- Pain management: the management of painful bone lesions poses a significant challenge in clinical practice, prompting researchers to explore various treatment modalities to alleviate pain and improve patients’ quality of life. Among these modalities, combined VP and RFA treatment, as well as VP alone, have garnered considerable attention for their potential efficacy in refractory cases.

Several studies, in fact, have compared the methods alone (either RFA or VP) and in combination. While a study by Orgera et al. shows no statistically significant difference between the two groups and that VP alone appears to be effective in pain management [27], other studies, particularly by Tian et al., suggest that the combination of the two methods yields better results, especially in pain management [29]. Indeed, the overall pain relief rate was significantly better in the group that used the combination. Similarly, the study group led by Wang et al. found that both VP alone and the combination of RFA and VP are safe and effective for the treatment of painful sacral metastases; the combination of the two techniques provide better pain control, which was more stable over time, and significative lower rates of venous cement leakage compared to using a single method [31]. Supporting these findings is the study by Yildizhan et al., which also highlights the fact that ablation combined with VP is more successful than VP alone for controlling palliative pain and preventing tumor spread in patients with painful vertebral metastasis [32].

In most studies employing RFA and VP, no serious complications were observed, although some minor complications such as transient neurological motor deficits and pulmonary embolism were reported, as noted by Nilgun Senol et al. [28] These positive results are also confirmed in studies involving vertebral pedicles [30].

Radiotherapy remains the leading treatment for spinal metastasis, providing pain relief. However, it has its limitations. These include a delay in the onset of pain control and the possibility of tumors being resistant to treatment. In these cases, pain relief is often a gradual process, with limited effectiveness. While some patients experience complete or partial pain reduction, success rates are not always optimal. Additionally, options for retreatment may be limited if pain recurs [54,55,56]. On the other hand, our review highlights the rapid onset of pain management with the combined ablative treatment with VP. As evidenced by the results, improvement is observed as early as the day after treatment, maintaining a good level in most cases over the subsequent weeks and months, and consistently better than the pre-treatment baseline.

- Tumor progression: Although studies focusing on local tumor control are fewer in number compared to those on pain management, it is evident that tumor control still yields optimal results, maintaining very low local recurrence rates, as demonstrated by the results of Yang et al., Tomasian et al., and Yildizhan et al. [14,16,32,34].

Furthermore, is widely demonstrated that, in addition to technical success and pain control, the complication rate is also low, making this combination of procedures quite safe. This is highlighted by Tomasian et al., with a total complication rate of 3% (of which only 0.4% were major complications) [14].

- Biomechanics, strength and mechanical stability: studies using samples in human cadaveric spines have examined how RFA and combined RFA and VP treatments affect the biomechanics of metastatic vertebrae. While RFA effectively targets tumors and causes cell death, it creates cavities in the vertebral body. These cavities can weaken the posterior wall, potentially leading to bulging and an increased risk of burst fractures under pressure. However, the study conducted by Pezeshki et al. shows that adding VP after RFA improves spinal stability, especially when the cement reinforces the posterior wall [46]. These findings highlight the importance of considering both ablation volume and cement distribution patterns to optimize vertebral stability after treatment.

### 4.2. MWA and VP

MWA represents a modern addition to the array of minimally invasive cancer treatments. Similar to thermal ablation techniques, MWA induces coagulation necrosis within the targeted tissue, leading to reduced production of nerve-stimulating cytokines and the destruction of pain nerve fibers in the periosteum and bone cortex. However, it is important to note that while MWA effectively addresses pain by ablating tissue, it does not contribute to enhancing the structural stability of the affected vertebral body. RFA and MWA operate through a distinct heating mechanism. While RFA heating may encounter limitations in tissues with low electrical conductivity, MWA offers rapid, efficient, and uniform heating without the risk of heat charring. These characteristics render MWA a suitable option for ablating bone tumors, particularly those of osteoblastic or mixed-density metastases [57].

Similarly to RFA combined with VP, studies also demonstrate the superiority of combined treatment for MWA. In a retrospective study by Liu et al., indeed, two groups were compared: one treated with VP alone and another with MWA combined with PV. The findings indicated that MWA combined with VP for painful spinal metastases achieved sustained pain relief (>6 months) and enhanced quality of life, while demonstrating lower rates of cement leakage and lower tumor progression compared to VP alone [24].

Furthermore, Chen et al. demonstrated promising outcomes regarding pain management and safety with the combination of MWA and VP. The technical success rate was 100%, with significant reductions observed in the median VAS score and mean post-procedure morphine dose compared to pre-procedural values at various follow-up time points, indicating sustained pain relief. Additionally, improvements were noted in the ODI score. Furthermore, a high rate of local control was achieved, with asymptomatic cement leakage observed in a minority of cases, suggesting not only the effectiveness but also the safety of the procedure [50].

### 4.3. RFA vs. MWA

RFA is recognized for its efficacy in treating osteolytic or mixed osteolytic–osteoblastic lesions, particularly those with minimal extraosseous involvement. However, RFA has limitations, including its restricted ablation volume, the necessity to penetrate the lesion, and the potential requirement for pads due to the high impedance encountered in cortical bone. Conversely, MWA offers advantages such as the ability to ablate sclerotic lesions effectively and achieve larger tumor ablation volumes within shorter timeframes. Nonetheless, MWA procedures may pose challenges in terms of procedural control [5].

In the literature, studies comparing MWA with VP and RFA with VP are rare. The multicentric study conducted by Alfonso et al. confirmed that the combination of ablation techniques with vertebral cementation is a safe approach, significantly enhancing patient pain relief and potentially aiding disease control. However, in terms of VAS scores post procedure and at 6 weeks, no significant difference was found between the efficacy of the two methods [26].

### 4.4. Risk of Bias

The studies included in this review were assessed for risk of bias using the ROBINS-I tool, focusing on several critical domains. A significant portion of these studies were retrospective, leading to a moderate risk of selection bias due to the lack of randomization in participant selection. This absence of randomization also contributed to a serious risk of bias in the classification of interventions, in many cases. However, there were some prospective studies included, which help to mitigate some of the biases typically associated with retrospective designs, though challenges related to blinding were still present. The measurement of outcomes was an area of concern, with a moderate risk of bias in many studies due to the lack of blinding of outcome assessors. The selection of reported results posed a moderate risk of bias, especially in the retrospective studies, where the potential for selective reporting existed. The risk of bias due to missing data varied, with some studies experiencing loss of follow-up data, particularly beyond the short- to mid-term periods, which introduced a serious risk in assessing long-term outcomes. In summary, while many of the studies included present biases, primarily related to their retrospective designs and lack of blinding, the inclusion of prospective studies provides a more balanced perspective. Nonetheless, the overall conclusions should be interpreted cautiously, considering the inherent limitations across the body of evidence.

### 4.5. Innovative Techniques and Devices

According to Pusceddu et al., navigational devices are favored for accessing challenging spinal metastases. In a study assessing the effectiveness of RFA and VP using a navigational radiofrequency ablation device for technically difficult posterior vertebral body metastatic lesions, they achieved 100% technical success. The mean VAS score dropped significantly from 5.7 to 0.9 post-RFA (*p* < 0.001), indicating immediate and durable pain relief. No local tumor progression or recurrence was observed during a median follow-up of 19 months for patients with 1–2 vertebral metastases and 10 months for those with multiple lesions. The navigational device enhanced precision and effectiveness in targeting these challenging lesions [35].

In more challenging cases, such as those requiring even more precise approaches, the transoral percutaneous approach combining ablation and cementoplasty may be necessary, as reported in a case report of a 40-year-old female with a solitary plasmacytoma of the right transverse apophysis of C1 [58].

Employing a steerable needle guided by a navigation system, in fact, enabled accurate targeting and access to a complex lesion. The capability of the distal segment of the ablation probe to flex up to 90° proved advantageous, especially in treating tumors located in challenging anatomical sites. This method allowed precise adaptation to the oral cavity and the specific morphology of the lesion, ensuring thorough coverage during ablation and maintaining adequate safety margins through a single bone-access channel.

Furthermore, Pusceddu et al. demonstrated, in a recent retrospective study, that combining MWA with SpineJack implants and VP for managing painful thoracolumbar pathological vertebral compression fracture is safe and effective. The treatment achieved 100% technical success, with significant pain reduction and vertebral height restoration, without major complications or recurrence, showing how this combined innovative technique could be a promising tool for these types of patients [53].

### 4.6. Emerging Frontiers and Artificial Intelligence

Artificial intelligence (AI) could be a promising tool for enhancing ablative and VP procedures, mirroring its successful application in other fields [59]. It might improve precision in targeting and ablating tumors, potentially increasing the efficacy of treatments. AI could also assist in real-time imaging and navigation, reducing the risk of complications and improving overall safety. Additionally, AI-driven data analysis can help identify optimal locations, leading to significant impacts on patient outcomes. It can also predict patient outcomes, personalize treatment plans, and monitor post-procedural recovery. By integrating AI into these procedures, healthcare providers could achieve higher accuracy, better patient outcomes, and more efficient workflows, making AI a valuable addition to the field of vertebral fracture treatment [59,60].

## 5. Conclusions

In conclusion, our systematic review unveils three pivotal observations that underscore the efficacy and safety of the combined RFA or MWA and VP intervention. Firstly, our analysis reveals a significant reduction in numerical pain scores among the subjects undergoing the combined treatment. This finding highlights the intervention’s effectiveness in alleviating pain, a critical aspect in enhancing the quality of life for individuals suffering from vertebral pathologies, and, in particular, highlights the rapid onset of pain relief provided by this combined treatment, which is faster compared to radiotherapy. Secondly, our review demonstrates the efficacy of the combined treatment in achieving local tumor control, coupled with low rates of tumor progression. This outcome underscores the therapeutic potential of RFA or MWA when integrated with VP, offering promising prospects for managing tumor growth and preventing disease progression. Lastly, our comprehensive assessment indicates a favorable safety profile associated with the combined intervention, characterized by low rates of both major and minor complications. This aspect is crucial in evaluating the overall risk–benefit balance of the treatment approach and ensuring patient safety throughout the therapeutic process. In summary, our systematic review provides compelling evidence supporting the efficacy, tumor control potential, and safety of the combined RFA or MWA and VP intervention. These findings warrant further investigation and potential integration into clinical practice, offering new avenues for improving patient outcomes in the management of vertebral pathologies.

## Data Availability

The data presented in this study are available in this article.

## Supplementary Materials

The following supporting information can be downloaded at: https://www.mdpi.com/article/10.3390/curroncol31090401/s1, Table S1: PRISMA 2020 Checklist. Reference [61] is cited in the Supplementary Materials.

## Abbreviations

MM	multiple myeloma
RFA	radiofrequency thermal ablation
VP	vertebroplasty
MWA	microwave ablation
IR	interventional radiology
PMMA	polymethylmethacrylate
VAS	Visual Analog Scale
ODI	Oswestry Disability Index
KPS	Karnofsky Performance Scale
NRS	Numeric Rating Scale
